# Using complaints from obstetric care for improving women’s birth experiences – a cross sectional study

**DOI:** 10.1186/s12884-023-06022-5

**Published:** 2023-10-03

**Authors:** Sisse Walløe, Søren Bie Bogh, Søren Fryd Birkeland, Lone Kjeld Pedersen, Annemette Wildfang Lykkebo, Lise Lotte Torvin Andersen, Britta Frederiksen-Møller, Lars Morsø

**Affiliations:** 1 OPEN Research Unit, Odense University Hospital, J. B. Winsløws Vej 9 a, 3rd floor, Odense C, 5000 Denmark; 2 The Research and Implementation Unit PROgrez, Physio- and Occupational Therapy Unit, Naestved-Slagelse- Ringsted Hospitals, Faelledvej 2c, Slagelse, 4200 Denmark; 3 Department of Clinical Research, University of Southern Denmark, J. B. Winsløws Vej 15, 3, Odense C, 5000 Denmark; 4 Department of Regional Health Research, University of Southern Denmark, J. B. Winsløws Vej 15, 2, Odense C, 5000 Denmark; 5 Gynecological Obstetric Unit D, Odense University Hospital, Kløvervaenget 5, Odense C, 5000 Denmark; 6 Women’s Health and Labour, Hospital of Lillebaelt, Sygehusvej 24, Kolding, 6000 Denmark; 7 Women’s Health and Labour, Hospital of Southern Jutland, Kresten Phillipsens Vej 15, Aabenraa, 6200 Denmark

**Keywords:** Healthcare complaints, Healthcare Complaints Analysis Tool (HCAT), Obstetric care, Relational issues, Individualized care, Continuity

## Abstract

**Introduction:**

Staff shortages and quality in obstetric care is a concern in most healthcare systems and a hot topic in the public debate that has centred on complaints about deficient care. However there has been a lack of empirical data to back the debate. The aim of this study was to analyse and describe complaints in obstetric care. Further, to compare the obstetric complaint pattern to complaints from women about other hospital services.

**Materials and methods:**

We used the Healthcare Complaints Analysis Tool to code, analyse and extract contents of obstetric complaint cases in a region of Denmark between 2016 and 2021. We compared the obstetric complaint pattern to all other hospital complaint cases in the same period regarding female patients at a large University Hospital in a cross-sectional study.

**Results:**

Complaints regarding obstetric care differed from women’s complaints regarding other healthcare services. Women from obstetric care raised more problems per complaint, and tended to complain more about relational issues indicated by odds for complaints about staff shortage four times higher in the obstetric care group. Women from obstetric care had a lower proportion of compensation claims.

**Conclusion:**

Systematic complaint analysis acknowledged women’s experience in obstetric care and may point to areas that potentially need further attention. Complaints from obstetric care show that women experience deficiencies related to relational problems like recognition and individualized support compared to complaints from women receiving other hospital healthcare services.

**Supplementary Information:**

The online version contains supplementary material available at 10.1186/s12884-023-06022-5.

## Introduction

In Denmark, there are 60.000 births annually. 97% of all births take place at the 23 maternity wards across the country [1] and Denmark is one of the safest countries to give birth in [1]. Despite this, Danish media have brought a series of traumatic birth stories, and public statements from midwives and obstetricians about high workload, poor birthing facilities, and organizationally challenged obstetric care depicting a severe crisis in the obstetric care services [2, 3]. In contrast, in a Swedish survey, only 3–6% of the participants reported negative birth experiences [4, 5], and the 2020 national Danish patient satisfaction survey found that 85% of the participating women were highly or very highly satisfied with care during child birth [6]. On an overall level, the satisfaction seems high [7], but several studies indicate that the satisfaction declines when detangling women’s birth experiences in detail [8, 9]. Perception of fear and birth experiences integrates patients’ life story, their care pathways and the maternity care system [10]. Women describe loss of control, helplessness, they feel unable to act adequate during childbirth and state that discrepancies between expectations and experiences affects their childbirth. The negative experiences leave psychological impressions for years after childbirth [11]. These experiences require high levels of staff support, and balanced information on expectations, to overcome [12, 13]. A 2021 study concludes that positive birth experiences contribute to parents feeling included, respected and safe [14]. Descriptions are in line with findings at a major hospital in Denmark’s Capital Region. Here women express that the attention and support they received during labor disappeared upon transfer to the post-natal wards [9, 15]. In the study lack of clinician continuity and staff shortages were central issues which may leave parents excluded and anxious, leading to a negative care experience [14]. Therefore, it appears that traumatic birth experiences are not necessarily only defined by somatic complications [9] but also by a lack of individualized care and continuity [11]. High workload and staff shortage are well-known predictors of patients experiencing substandard care that can lead to filing complaints [12, 16]. By using filed complaints as data source we are able to review the unfiltered experiences from individuals who, from their point of view, have had a negative birth experience.

One could, therefore, hypothesize staff- and resource shortage to be a common problem mentioned in complaints regarding obstetric care. But it is unclear if clinician continuity and staff shortages are of a magnitude that triggers complaints or if potential complaint cases contain other problem areas. It is also unclear whether the patterns regarding negative birth experiences can be generalized to other patient groups across settings, though comparison might be difficult. Nonetheless, the questioning of obstetric care quality has entailed public demands for improved quality in obstetric care. Though potentially powerful, the public demands seem unstructured and not taking empirical basis or underlying data into account.

We wish to investigate the complaint pattern for obstetric hospital care and compare this to the general complaint pattern for female patients in the age group 16 to 45 years receiving other healthcare services at hospitals in a Region of Denmark.

## Materials and methods

### Setting

In Denmark, obstetric care is publically funded. Secondary sector obstetric care is mostly managed by 25 hospitals in the five Danish Regions. Home births constitute 3.1% of births (2016) according to the Danish Health Authority. Primi-parous women are offered 24 h maternity care at most hospitals.

In the Region of Southern Denmark, Odense University Hospital (OUH) is the largest hospital with approx. 1.100.000 contacts a year (submitted and out-patients). Its obstetric care is delivered across 2 locations, with approximately 4,700 births every year, of which 800 low-risk births are at the smaller birth unit of OUH, Svendborg. The region’s other three obstetric care units are at the Hospital of Lillebaelt (SLB) (3,300 births p.a.), Hospital of Southern Jutland (SHS) (1,750 births p.a.), and Hospital of South West Jutland (SVS) (1,800 births p.a.). SLB and SHS handle normal and complicated births from gestational age (GA) 28 weeks, SVS from GA 32 weeks. High-risk births are referred to OUH.

### Material and data sources

This cross-sectional study included complaints about obstetric care from a sample of women from GA 22 + 0 weeks to 2 weeks postpartum giving birth at one of the included hospitals in the region. Obstetric care is defined as antenatal care from 22 + 0 weeks and midwifery visits in secondary care, care during labor, and postnatal care. The 22 + 0 weeks period was chosen to fit the Danish Health authorities’ definition of giving birth [1]. The 2 weeks postpartum demarcation is to separate complaints concerning obstetric care from other care on e.g. neo-natal wards. To have an appropriate sample size we included complaints and compensation claims about obstetric care at OUH, SLB or SHS filed between Jan 1, 2016, and Jun 30, 2021. We manually checked all files to assure that we only included cases regarding births. We did not distinguish between complicated and uncomplicated births or vaginal and C-section births.

In Denmark patients can file a ‘non-monetary’, disciplinary complaint about unsatisfactory health care and/or file a compensation claim. Disciplinary complaints are assessed by health specialists appointed with the Danish Health Complaints and compensation claims at the Patient Assurance Organization.

Complaints are filed either locally at the healthcare provider (e.g. at the hospital or ward) or to the National Complaint Authority (NCA) (www.stpk.dk) [17]. Compensation claims are filed with the Danish Patient Insurance Association (DPIA) (www.patienterstatningen.dk). Complaints are usually filed by the patient or a relative, but can in rare cases be filed by a third party (e.g. a patient attorney or a clinician). This study includes NCA complaints, compensation claims filed with the DPIA, and complaints about the level of service, waiting time, etc. filed directly with the obstetric care unit or hospital administration, but for the sake of ease they are all referred to as ‘complaints’ in the text. The complaints had to be filed within the inclusion period (Jan 1, 2016, and Jun 30, 2021), but could potentially have happened before this period as there is no limitation on when to file a complaint.

To compare the patterns across diagnosis and settings we compared the complaint cases from women from obstetric care to a sample of healthcare complaints filed by female patients aged 16 to 45 years journalized at OUH between 2016 and 2020. We chose this age group to reflect the age of most women from obstetric care. In this comparison group we excluded all obstetric cases, but included the remaining complaints regarding treatment across diagnosis (e.g. orthopaedics, cancers and contacts at the emergency department), aware of the fact that the comparison group was heterogeneous.

When patients file a complaint to the NCA, they indicate whether they want a dialogue regarding their complaint. If so, the hospital are required to facilitate a dialogue, evaluate it, and report to the authorities. If the patients do not want a dialogue, it is noted in the patient record but it does not affect the processing of the complaint. For complaints filed about obstetric care, it was recorded if a dialogue was held and the outcome of these dialogues.

### Complaint contents categorization

Complaint letters were coded using the Danish version of Health Complaint Analysis Tool (HCAT) [18]. The HCAT has been shown suitable for systematic and reliable analysis of health care compensation claims in a Danish setting [19]. The HCAT taxonomy condenses all problems mentioned in complaint letters into three domains (Clinical, Management, and Relationships) and seven problem categories (Quality, Safety, Environment, Institutional processes, Listening, Communication, and Respect and Patient rights). The identified problems are further divided into 36 sub-problem categories (see supplementary material). The HCAT assesses sub-problem severity, the stage of care at which the complaint occurred, and the overall harm caused. Severity addresses the potential risk of the problem, where harm relates to the actual harm experienced by the patient. It is noted who filed the complaint (the patient, relative or others), the gender of the patient, and which staff groups were involved in the incident [18, 20]. Complaint letters may mention several problems, which are all coded according to the HCAT taxonomy (Table 1).

**Table 1:** The HCAT Classification system

Domain	Problem category	Sub-categories *Each rated from 0 (not evident) to 3 (high severity)*
Clinical	Quality	Neglect (3)*: Hygiene & personal care; Nourishment & hydration; General Rough handling & discomfort Examination & monitoring Making & following care plans Outcomes & side effects
	Safety	Error (3)*: Diagnosis; Medication; General Failure to respond; Clinician skills; Teamwork
Management	Environment	Accommodation; Preparedness; Ward cleanliness; Equipment; Staffing; Security
	Institutional processes	Delay (3)*: Access; Procedure; General Bureaucracy; Visiting; Documentation.
Relationships	Listening	Ignoring patients; Dismissing patients; Token listening
	Communication	Delayed communication; Incorrect communication; Absent communication
	Respect and patient rights	Disrespect; Confidentiality; Rights; Consent; Privacy
Stages of care	(1) Admissions, (2) examination & diagnosis, (3) Care on the ward, (4) Operations & procedures, (5) Discharge & transfers, (6) Unspecified/other	
Levels of harm	Experienced by the claimant: *rated from 0 (none/no information about harm) to 5 (catastrophic harm)*	

* There are three types of; ’Neglect’, ’Error’ and ’Delay’

### Analysis

The systematically coded data from the complaints were entered into a REDCap (Research Electronic Data Capture) database. We used descriptive statistics (numbers, proportions and inter quartile ranges (IQR)) for distributions and used non-parametric statistics (Pearson’s rank and chi-squared test) for comparison. We used logistic regression (odds ratios) to calculate associations across the obstetric complaint cases and the filed complaints from the included control group. We calculated the proportion of held dialogues by patient reported harm in the obstetric complaint cases. All data management and analyses were performed in Stata, version 15 (StataCorp LP, College Station, Texas).

### Ethics

The study was carried out in accordance with relevant guidelines and regulations. According to Danish law, research based on register data requires no approval from research ethics committees and no informed consent for the use of data is needed. However, it must satisfy general data protection regulation (Directive 95/46/EC; 2016/679 and DK Act 502). The research protocol was approved and we obtained permission for review of complaint cases and for storage and analysing data (journal number20/31,504) from the local ethics committee at the University Hospital Odense.

## Results

Our study included 216 obstetric complaint cases and 759 complaints regarding other hospital services for female patients in the age group 16 to 45 years. Patients dissatisfied with obstetric care were significantly less likely to file a compensation claim (45.4% obstetric cases vs. 75.8% other hospital cases, OR 0.3 [CI 95% 0.2; 0.4]) but significantly more likely to file a complaint with the Patient Complaint Authority (44.4% obstetric cases vs. 16.9% other hospital cases, OR 3.9 [CI 95% 2.8; 5.5]). In contrast to other hospital care where complaints were mainly filed against physicians, complaints concerning obstetric care were equally directed at physicians and other medical staff (i.e. midwives) (Table 2).

**Table 2:** Obstetric complaint cases and all other hospital complaint cases filed by women (age 16–45)

	Obstetric care	Other hospital care	*p*-value
Total complaint cases	216^	759^^	
Complaint case type, n (%)			< 0.001
- Service	6 (2.8)	26 (3.4)	
- Compensation claim	98 (45.4)	575 (75.8)	
- Complaint	96 (44.4)	128 (16.9)	
- Missing	16 (7.4)	30 (4.0)	
Who filed the complaint, n (%)			0.12
- Family member	32 (14.8)	56 (7.4)	
- Patient	178 (82.4)	667 (87.9)	
- Unspecified/Other	6 (2.8)	36 (4.7)	
Staff groups the complaint refer to^†^, n (%)			
- Administrative	9 (4.2)	20 (2.6)	
- Physicians	137 (63.4)	651 (85.8)	
- Nursing and other medical staff (i.e. midwives)	143 (66.2)	82 (10.8)	
- Unspecified/Other	17 (7.9)	97 (12.8)	

^ obstetric complaint cases at OUH, SLB, SHS in the period of 1/1 2016 to 31/6 2021

^^ complaint cases regarding all other hospital at OUH services in the period of 1/1 2016 to 31/12 2020

† Each complaint case could refer to multiple staff groups. No group p-value was calculated

Overall, the obstetric complaint cases had a median of 3 problems per case displaying 728 problems in 216 cases compared to 2 problems (1552 problems in 759 cases) in the overall comparison group. For both groups, the problem categories ‘quality’ and ‘safety’ were most commonly reported, but still ‘quality’ and ‘safety’ issues were less frequent in obstetric complaint cases (31% and 20% in obstetric cases vs. 39% and 23% in other hospital cases). In contrast, there were slightly more complaint problems recorded in the main categories of ‘listening’ and ‘respect/patient rights’ for the obstetric group compared to the overall hospital group (14% and 10% vs 11% and 7%, respectively), and in the category ‘environment’ with 9% vs 3% in the overall hospital population. Complaints reported during ‘care on the ward’ were almost four times as common in obstetric care compared to complaints about other hospital care incidents (28% vs. 8%, respectively)(Table 3).

Although the severity of problems experienced by patients was similar for obstetric and overall hospital complaint cases reported, there were some differences in the reported harm. 41% of the obstetric problems were almost equally related to complaints either reporting major (19%) or catastrophic harm (22%), whereas this proportion was 56% for overall hospital population with the majority of problems being major (40%). The proportion of moderate harm reported in obstetric complaints was higher than for overall hospital complaints (36% vs. 24%), while complaints reporting minor or minimal harm were similar across the two groups (21% vs. 18%) (Table 3).

**Table 3:** Complaint categories, stages of care, severity and harm* in obstetric care vs. other hospital services^

	Obstetric care	Other hospital care	*p*-value
Total number of complaint problems in the filed complaints, n			
	728	1,552	
Complaint problems per case, median [IQR]	3; [1;5]	2 [1;2]	< 0.001
Problem categories, n (%)			< 0.001
• Quality	225 (30.9)	608 (39.2)	
• Safety	147 (20.2)	359 (23.1)	
• Environment	67 (9.2)	51 (3.3)	
• Institutional processes	52 (7.1)	134 (8.6)	
• Listening	105 (14.4)	172 (11.1)	
• Communication	62 (8.5)	117 (7.5)	
• Respect and patient rights	70 (9.6)	111 (7.2)	
Stages of care for complaint problem items, n (%)			< 0.001
• Admission	39 (5.4)	49 (3.2)	
• Examination/diagnosis	179 (24.6)	453 (29.2)	
• Care on ward	207 (28.4)	120 (7.7)	
• Operation/procedures	213 (29.3)	595 (38.4)	
• Discharge/transfers	29 (4)	33 (2.1)	
• Other/unspecified	11 (1.5)	74 (4.8)	
• Missing	50 (6.9)	228 (14.7)	
Severity, n (%)^†^			0.64
• Low	144 (19.8)	285 (18.4)	
• Medium	365 (50.1)	807 (52.0)	
• High	219 (30.1)	460 (29.6)	
Harm, n (%)^†^			< 0.001
• Minimal	56 (7.7)	143 (9.2)	
• Minor	100 (13.7)	130 (8.4)	
• Moderate	263 (36.1)	375 (24.2)	
• Major	140 (19.2)	614 (39.6)	
• Catastrophic	161 (22.1)	255 (16.4)	
• N/A	8 (1.1)	35 (2.3)	

* Using the HCAT taxonomy for coding

^ For women in the age of 16 to 45 years

† Level of harm relates to the outcome as described by the patient, and severity relates to the potential hazard of a given problem independent of actual harm

Sub analyses of problems in the ‘environment’ category showed that the proportion for complaining about staff shortages was more than four times higher for the obstetric group than for the overall hospital group (3.6% vs. 0.5%, respectively). The proportion of complaints in all sub-categories of the ‘listening’ category (‘ignoring patients’, ‘dismissing patients’, and ‘token listening’) were higher in the obstetric group. Further, most complaints within the sub-category ‘patient rights’ were more frequent for obstetric cases (Table S1).

**Fig. 1:**
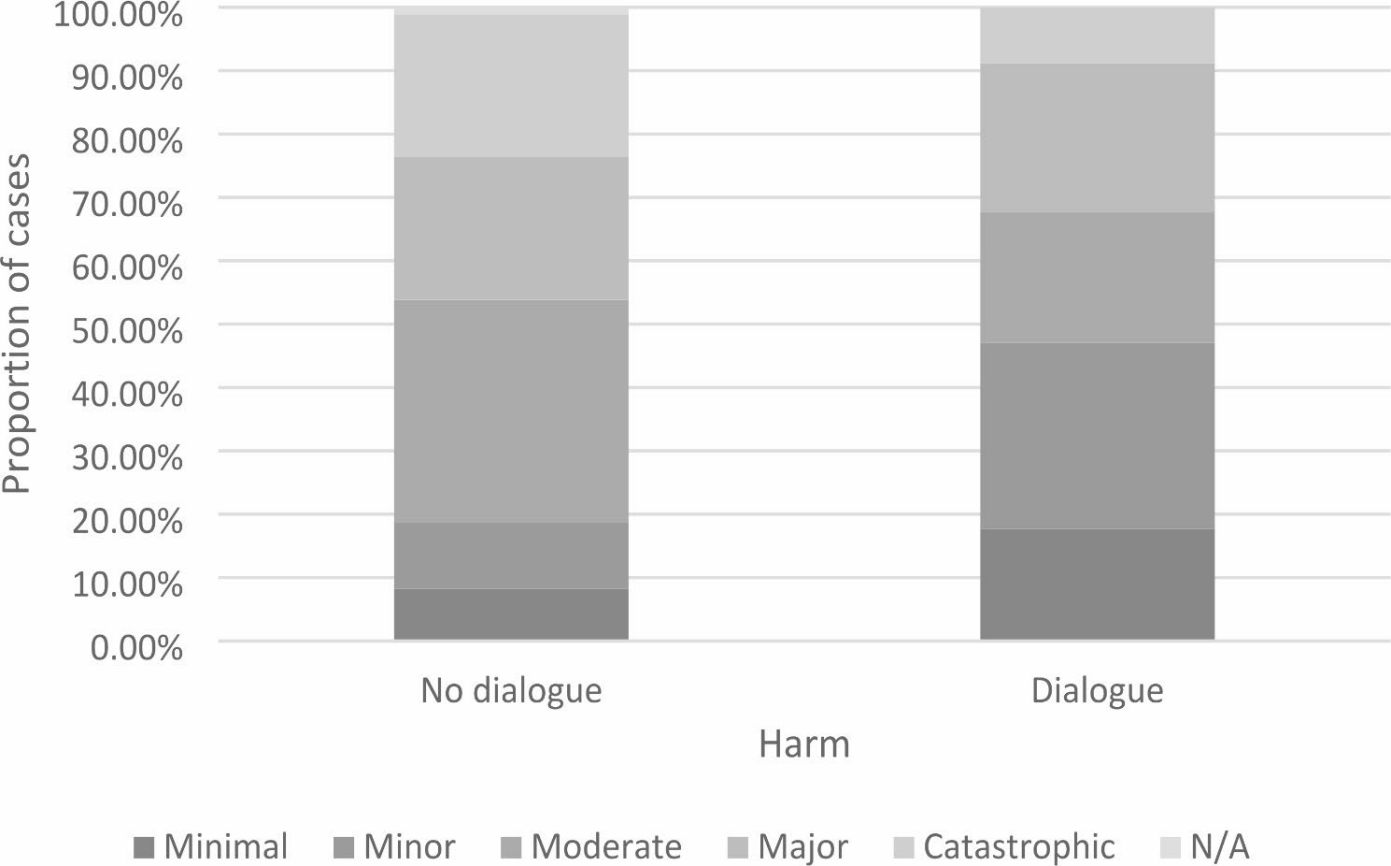
Completed dialogues by patient reported harm for women filling an obstetric compliant case

Ninety six obstetric complainants were entitled to a dialogue, but only 34 dialogues were held. Dialogues were more likely to be held if the complaint contained problems in the categories ‘listening’, ‘communication’, and ‘respect’ (figures not shown), and the proportion of cases not having dialogue were higher if catastrophic harm was reported (Fig. 1).

## Discussion

In this study, we wanted to investigate the complaint pattern for obstetric hospital care in the Region of Southern Denmark and further compare this to the general complaint pattern for female patients in the age group 16 to 45 years receiving all other kinds of healthcare services at a single hospital in the same region.

Overall we only found a small proportion of complaints for both obstetric and general healthcare services, but in the obstetric care group we found significantly more complaint problems per filed complaint. We realize that the comparison between women treated for diseases that range from light injuries to deadly diseases and healthy women who give birth to, in most cases, a healthy child might seem somewhat arbitrary and could be questioned as comparing apples and pears. Still our findings give rise to thought. One might think that women giving birth would complain frequently, but this does not seem to be the case. Some women experience postpartum haemorrhage and sphincter injury [4] and though the outcome of child birth mostly turns out fine, these negative birth experiences leave impressions in women for decades [11] and might lead to filing a complaint. Further, not having to deal with long-term illness, might give the women in obstetric care mental surplus to complain while women who have survived major illness, in contrast, might have a higher threshold for complaining.

We also found that obstetric complaint cases differed from cases regarding women receiving other healthcare services at the hospitals OUH, SLB, and SHS. Though the complaints about obstetric care contained more problems. Obstetric cases contained fewer problems related to the ‘quality’ and ‘safety’ area than general cases, but more problems about ‘listening’, ‘respect’ and ‘environment’. A variety of risk factors affect the experience of giving birth, such as; unexpected problems during child birth, feelings during labour (e.g. unexpected pain level or lack of control), lack of time allocated resulting in less clinician support, discrepancy between information received during the pregnancy and the actual child birth, lack of follow up after the birth [7]. These factors reflect the problem areas of ‘listening’, ‘respect’ and ‘environment’ and could explain the overrepresentation of complaints in these areas. The consequences of complaints regarding this dimension should not be underestimated and associations of affecting future reproduction have been reported [21].

Even though the severity of complaints about obstetric care was comparable to the severity of other healthcare services complaints, women in obstetric care experienced overall less harm. The experience of less harm by the women in obstetric care reflects Denmark being one of the safest countries to give birth in [1]. WHO state that birth related deaths have decreased over the last twenty years with five deaths per 100.000 births. In line with this, the women dissatisfied with their obstetric care were less likely to claim compensation.

It has previously been shown that complications and obstetric interventions are associated with dissatisfaction and this is also the case for women being induced [4]. Those women experience staff shortage, neglect, and increased anxiety and, therefore, suggest increased focus during the delivery process [22]. It could be speculated that time pressure and staff shortages in a busy health care system result in too much workload, which could be one explanation of the particular complaint pattern revealed in this study regarding relational problems. These are typically problems that, fortunately, have a tendency to cause less harm, but over time problems relating to relational and continuity issues could create a culture with increased pressure for having i.e. induction due to time pressure leaving less room for an optimized birth experience and a risk for increased complaints.

Promotion of positive birth experiences is related to a safe environment, including trustful relationships [23]. The higher occurrence of obstetric care complaints regarding ‘listening’ and ‘respect’, support the statement that some women experience a lack of recognition and individualized support in obstetric care at Danish hospitals. Our data partly adds support to the significance of environmental factors in obstetric care complaints by showing obstetric complaints often relate to staff shortages (but not to the physical environment).

Women giving birth are mostly healthy people and Denmark is one of the safest places to give birth in. As such, we may expect fewer obstetric patients to experience major or catastrophic harm than general patients, who may have been acute or severely ill when entering the hospital. Still, women who experience a traumatic birth do not necessarily have physical or psychological adverse outcomes [24]. A good birth experience may rather be linked to having a coherent birth narrative [25]. Hence, quality of obstetric care may amount to more than leaving the hospital as a medically healthy individual with a healthy child.

Highlighting these complaint patterns based on patients’ experiences with health care services contributes to the empirical base and adds evidence to the ongoing discussion of quality in health care overall and in obstetric care in particular. Our finding might help to qualify the discussion on potential organizational changes in the future.

### Strengths and limitations

This study only had access to journalized complaints; hence there may be complaints (e.g. by e-mail or phone) that are not included in this material but from personal contact with administrative staff who handles complaints only very few complaints are not journalized. Further, we were not able to collect complaint data from all hospitals in the region. Therefore, we collapsed obstetric complaint data from three regional sites, and compared it to overall complaint data that only was available from OUH. We have no reason to believe that including the remaining two sites would have changed the obstetric data. In contrast, using overall comparison data might have resulted in a higher proportion of compensation claims with OUH being a university hospital with highly specialized functions.

In this study we did not have access to individual patient records. Therefore, we have no knowledge of who had instrumental deliveries or emergency caesarean sections. It could be argued that complicated deliveries and perceived ill health before and during pregnancy increases the likelihood of filing a complaint. We do not know if that is the case, but we are in doubt if these are factors that could explain the higher proportion of complaints compare to other hospital services.

It is well known that risk of substandard healthcare services among disadvantaged exist [26, 27]. In contrast, only little is known on social inequality when filing a complaint when experiencing suboptimal care. An earlier study on inequality in applying for compensation after acute hospital services found some inequality in compensation claims and compensation payments regarding acute healthcare services [28]. It is likely that inequality in filing a complaint after having experienced suboptimal care also exists and further research to pursue this has been planned.

Although it is stated that officially reported complaint cases only represent “the tip of the iceberg” when investigating sub-standardized healthcare services, the number of complaints are increasing [29]. Currently, we lack methods to capture the complete overview of the prevalence of traumatizing, unsatisfactory obstetric care experiences. In future studies, data from adverse events could be a relevant source to explore further, but this requires a systematic approach in line with the complaint cases.

The number of recorded dialogues was relatively sparse and may not reflect the actual number of dialogues. We would have liked to present how many women declined having a dialogue about their obstetric complaint, but we did not have access to these data.

Care during pregnancy, childbirth, and the puerperium involves several consultations and several persons. We chose to focus on complaints about obstetric care in public hospitals, but in the future complaint material from primary healthcare services should be included.

Every single complaint from obstetric care have been viewed by the first author to ensure that the complaint had focal point within child birth, and though we do not have any further description of the delivery, we are sure that complaints from other areas of obstetric care were not included in the analysis.

## Conclusion

We found that complaints regarding obstetric care differed from complaints from women regarding other health care services. The proportion of complaints was higher and women who complained about obstetric care raised more complaint problems per complaint case. Further they complained more about ‘listening’, ‘respect’ and ‘environment’ with complaints specifically pointing at staff shortage. Our findings indicated a patient reported lack of recognition and individualized support in obstetric care at the hospitals. Highlighting complaint patterns based on patients’ experiences might help to qualify the discussion on potential organizational changes in the future.

### Electronic supplementary material

Below is the link to the electronic supplementary material.

Supplementary Material 1

## Data Availability

The data can be made available in anonymised form, but is current considered as personal data as is can be referred to individualized complaint case id and therefore compromise privacy. Data is currently journalized at the Odense University Hospital. The datasets used in the current study can be made available from the corresponding author on reasonable request.
