# Correction: Using complaints from obstetric care for improving women’s birth experiences – a cross sectional study

**DOI:** 10.1186/s12884-024-06291-8

**Published:** 2024-01-31

**Authors:** Sisse Walløe, Søren Bie Bogh, Søren Fryd Birkeland, Lone Kjeld Pedersen, Annemette Wildfang Lykkebo, Lise Lotte Torvin Andersen, Britta Frederiksen-Møller, Lars Morsø

**Affiliations:** 1 OPEN Research Unit, Odense University Hospital, J. B. Winsløws Vej 9 a, 3rd floor, Odense C, 5000 Denmark; 2 The Research and Implementation Unit PROgrez, Physio- and Occupational Therapy Unit, Naestved-Slagelse- Ringsted Hospitals, Faelledvej 2c, Slagelse, 4200 Denmark; 3 Department of Clinical Research, University of Southern Denmark, J. B. Winsløws Vej 15, 3, Odense C, 5000 Denmark; 4 Department of Regional Health Research, University of Southern Denmark, J. B. Winsløws Vej 15, 2, Odense C, 5000 Denmark; 5 Gynecological Obstetric Unit D, Odense University Hospital, Kløvervaenget 5, Odense C, 5000 Denmark; 6 Women’s Health and Labour, Hospital of Lillebaelt, Sygehusvej 24, Kolding, 6000 Denmark; 7 Women’s Health and Labour, Hospital of Southern Jutland, Kresten Phillipsens Vej 15, Aabenraa, 6200 Denmark

**Correction:** ***BMC Pregnancy Childbirth*** **23, 705 (2023)**

10.1186/s12884-023-06022-5

Following publication of the original article [1], the authors reported an error in Tables 2 and 3. The numbers are placed wrongly or incorrectly aligned. The correct tables are given below.

**Table 2:** Obstetric complaint cases and all other hospital complaint cases filed by women (age 16–45)

	Obstetric care	Other hospital care	*p*-value		
Total complaint cases	216^	759^^			
Complaint case type, n (%)			< 0.001		
- Service - Compensation claim	6 (2.8) 98 (45.4)	26 (3.4) 575 (75.8)			
- Complaint	96 (44.4)	128 (16.9)			
- Missing	16 (7.4)	30 (4.0)			
Who filed the complaint, n (%)			0.12		
- Family member	32 (14.8)	56 (7.4)			
- Patient	178 (82.4)	667 (87.9)			
- Unspecified/Other	6 (2.8)	36 (4.7)			
Staff groups the complaint refer to†, n (%)					
- Administrative	9 (4.2)	20 (2.6)			
- Physicians	137 (63.4)	651 (85.8)			
- Nursing and other medical staff (i.e. midwives)	143 (66.2)	82 (10.8)			
- Unspecified/Other	17 (7.9)	97 (12.8)			

^ obstetric complaint cases at OUH, SLB, SHS in the period of 1/1 2016 to 31/6 2021

^^ complaint cases regarding all other hospital at OUH services in the period of 1/1 2016 to 31/12 2020

† Each complaint case could refer to multiple staff groups. No group *p*-value was calculated

**Table 3:** Complaint categories, stages of care, severity and harm* in obstetric care vs. other hospital services^

	Obstetric care	Other hospital care	*p*-value	
Total number of complaint problems in the filed complaints, n	728	1,552		
Complaint problems per case, median [IQR]	3; [1;5]	2 [1;2]	< 0.001	
Problem categories, n (%)			< 0.001	
• Quality	225 (30.9)	608 (39.2)		
• Safety	147 (20.2)	359 (23.1)		
• Environment	67 (9.2)	51 (3.3)		
• Institutional processes	52 (7.1)	134 (8.6)		
• Listening	105 (14.4)	172 (11.1)		
• Communication	62 (8.5)	117 (7.5)		
• Respect and patient rights	70 (9.6)	111 (7.2)		
Stages of care for complaint problem items, n (%)			< 0.001	
• Admission	39 (5.4)	49 (3.2)		
• Examination/diagnosis	179 (24.6)	453 (29.2)		
• Care on ward	207 (28.4)	120 (7.7)		
• Operation/procedures	213 (29.3)	595 (38.4)		
• Discharge/transfers	29 (4)	33 (2.1)		
• Other/unspecified	11 (1.5)	74 (4.8)		
• Missing	50 (6.9)	228 (14.7)		
Severity, n (%)†			0.64	
• Low	144 (19.8)	285 (18.4)		
• Medium	365 (50.1)	807 (52.0)		
• High	219 (30.1)	460 (29.6)		
Harm, n (%)†			< 0.001	
• Minimal	56 (7.7)	143 (9.2)		
• Minor	100 (13.7)	130 (8.4)		
• Moderate	263 (36.1)	375 (24.2)		
• Major	140 (19.2)	614 (39.6)		
• Catastrophic	161 (22.1)	255 (16.4)		
• N/A	8 (1.1)	35 (2.3)		

* Using the HCAT taxonomy for coding

^ For women in the age of 16 to 45 years

† Level of harm relates to the outcome as described by the patient, and severity relates to the potential hazard of a given problem independent of actual harm

The original article has been corrected.
