# Supplementary material for: Using complaints from obstetric care for improving women’s birth experiences – a cross sectional study
Source: BMC Pregnancy Childbirth. 2023 Oct 3;23:705. doi: 10.1186/s12884-023-06022-5 (PMC10546670; doi:10.1186/s12884-023-06022-5)
Supplement: Supplementary file 1 — Supplementary Material 1 [file 12884_2023_6022_MOESM1_ESM.docx]

|  | Obstetric complaints | Other hospital complaints^ | | |
| --- | --- | --- | --- | --- |
| Complaint points | **N=728** | | **N=1,552** | |
| Quality: Clinical standards of healthcare staff behavior, n (%) | | | | |
| Neglect - Hygiene and personal care | 1 (0.1) | | 4 (0.3) | |
| Neglect - Nutrition and hydration | 7 (1.0) | | 6 (0.4) | |
| Neglect - General | 34 (4.7) | | 13 (0.8) | |
| Rough handling and discomfort | 32 (4.4) | | 53 (3.4) | |
| Examination and monitoring | 42 (5.8) | | 92 (5.9) | |
| Making and following care plan | 45 (6.2) | | 96 (6.2) | |
| Outcome and side effects | 64 (8.8) | | 344 (22.2) | |
| Safety: Errors, incidents, and staff competencies, n (%) | | | | |
| Error - Diagnosis | 42 (5.8) | | 182 (11.7) | |
| Error - Medication | 9 (1.2) | | 24 (1.5) | |
| Error - General | 49 (6.7) | | 54 (3.5) | |
| Failure to response | 6 (0.8) | | 24 (1.5) | |
| Clinician skills | 37 (5.1) | | 49 (3.2) | |
| Teamwork | 4 (0.5) | | 25 (1.6) | |
| Safety - other | 0 (0.0) | | 1 (0.1) | |
| Safety: Errors, incidents, and staff competencies, n (%) | | | | |
| Accommodation | 3 (0.4) | | | 0 (0.0) |
| Preparedness | 6 (0.8) | | | 3 (0.2) |
| Ward cleanliness | 1 (0.1) | | | 1 (0.1) |
| Equipment | 5 (0.7) | | | 4 (0.3) |
| Staffing | 26 (3.6) | | | 8 (0.5) |
| Safety | 1 (0.1) | | | 0 (0.0) |
| Infection | 25 (3.4) | | | 35 (2.3) |
| Institutional Processes: Problems in bureaucracy, waiting times, and accessing care, n (%) | | | | |
| Delay - Availability | 13 (1.8) | | | 21 (1.4) |
| Delay - Procedures | 16 (2.2) | | | 35 (2.3) |
| Transitions | 3 (0.4) | | | 8 (0.5) |
| Delay - General | 1 (0.1) | | | 22 (1.4) |
| Bureaucracy | 8 (1.1) | | | 18 (1.2) |
| Visiting | 1 (0.1) | | | 2 (0.1) |
| Documentation | 6 (0.8) | | | 9 (0.6) |
| Continuity | 1 (0.1) | | | 8 (0.5) |
| Prioritization | 3 (0.4) | | | 11 (0.7) |
| Listening: Healthcare staff disregard or do not acknowledge information from patients, n (%) | | | | |
| Ignoring patients | 28 (3.8) | | | 51 (3.3) |
| Dismissing patients | 52 (7.1) | | | 79 (5.1) |
| Token listening | 25 (3.4) | | | 42 (2.7) |
| Communication: Absent or incorrect communication from healthcare staff to patients, n (%) | | | | |
| Delayed communication | 12 (1.6) | | | 11 (0.7) |
| Incorrect communication | 24 (3.3) | | | 38 (2.4) |
| Absent communication | 21 (2.9) | | | 63 (4.1) |
| Absent family involvement | 4 (0.5) | | | 5 (0.3) |
| Language difficulties | 1 (0.1) | | | 0 (0.0) |
| Respect and patient rights: Disrespect or violations of patient rights by staff, n (%) | | | | |
| Disrespect | 35 (4.8) | | | 52 (3.4) |
| Confidentiality | 4 (0.5) | | | 6 (0.4) |
| Rights | 15 (2.1) | | | 16 (1.0) |
| Rights - Guarantee | 8 (1.1) | | | 23 (1.5) |
| Consent | 7 (1.0) | | | 12 (0.8) |
| Privacy | 1 (0.1) | | | 2 (0.1) |

Table S1. HCAT sub-categories* across complaints of obstetric care and other hospital services

* Data are presented as frequency and percentage (n, %).

^ For women in the age of 16 to 45 years.
